# Antioxidant Status, Blood Constituents and Immune Response of Broiler Chickens Fed Two Types of Diets with or without Different Concentrations of Active Yeast

**DOI:** 10.3390/ani12040453

**Published:** 2022-02-12

**Authors:** Youssef A. Attia, Hanan Al-Khalaifah, Hatem S. Abd El-Hamid, Mohammed A. Al-Harthi, Salem R. Alyileili, Ali A. El-Shafey

**Affiliations:** 1 Agriculture Department, Faculty of Environmental Science, King Abdulaziz University, P.O. Box 80208, Jeddah 21589, Saudi Arabia; malharthi@kau.edu.sa; 2 Environment and Life Sciences Research Center, Kuwait Institute for Scientific Research (KISR), P.O. Box 24885, Safat-Kuwait 13109, Kuwait; 3 Poultry and Fish Diseases Department, Faculty of Veterinary Medicine, Damanhour University, Damanhour 22511, Egypt; drhatem_deltavet@yahoo.co.uk; 4 Department of Integrative Agriculture, College of Food and Agriculture, United Arab Emirates University, Al-Ain 15551, United Arab Emirates; salem7178767@gmail.com; 5 Animal and Poultry Production Department, Faculty of Agriculture, Damanhour University, Damanhour 22511, Egypt; alielshafey2015@gmail.com

**Keywords:** active yeast, antioxidant status, broilers, nutrient density, immune response

## Abstract

**Simple Summary:**

Rations for broilers can be safely supplemented with probiotics such as active *Saccharomyces cerevisiae* (SC) yeast to stimulate oxidative reactions and immune response against stress and infectious agents. The current study suggested that SC yeast enhanced antioxidant capacity, growth rate, immune organ weights, immune response and the survival rate of broilers after Avian Influenza virus challenge at 38 days of age.

**Abstract:**

Probiotics, such as active yeasts, are widely used to enhance poultry production and reduce feeding costs. This study aimed to investigate the antioxidant and immune responses of broilers to different concentrations of active *Saccharomyces cerevisiae* (SC) when supplemented to two types of diets. A total of 216 1-day-old Arbor Acres unsexed chicks were used in a factorial design, involving two feeds (regular- versus low-density diet) and three concentrations of SC (0%, 0.02% and 0.04%). The results revealed that the low-density diet reduced the body weight and production index of broilers. The addition of SC improved the production index more than the control diet. Total antioxidant capacity (TAC), alanine aminotransferase (ALT), aspartate aminotransferase (AST) and eosinophils were significantly higher in response to the regular-density diet than the low-density diet; however, phagocytic activity (PA), lymphocyte and lysozyme activity (LYS) were lower. *Saccharomyces cerevisiae* reduced ALT, AST, malondialdehyde (MAD) and TAC more than the standard set, but improved packed cell volume (PCV), hemoglobin (Hgb), red blood cells (RBCs), lymphocytes, monocytes, heterophils, phagocytic index (PI) and the immune response to Newcastle disease virus (NDV) and avian influenza (AI). In conclusion, supplementation of a regular- or low-density diet with SC at a concentration of 0.02% or 0.04% improved the antioxidant parameters, immune status and production index of broilers against stress and infectious agents.

## 1. Introduction

There is a growing interest in the innovative biofortification of poultry feed rations through the use of functional ingredients to improve feed utilization and enhance production performance and the immune status of the flocks [1,2,3,4,5,6,7,8].

*Saccharomyces cerevisiae* (SC) has been used in poultry feed rations to enhance resistance to aflatoxicosis in poultry [9]. The antioxidant status and capacity of poultry have been shown to be significantly enhanced by supplementing poultry feed rations with SC, either alone or in combination with probiotics [10,11]. The weights of the primary and secondary immune organs in broilers have been shown to be increased after dietary supplementation with active yeast, potentially indicating immunocompetence in broilers [12]. In addition, Kiarie et al. [13] revealed that when added with feed enzymes, yeast derivatives can modulate cellular- and humoral-mediated immunity in broilers against intestinal coccidial infections. Zhou et al. [14] investigated the ability of yeast fractions to prevent pullorum disease and fowl typhoid in breeders. The results of the study revealed that dietary fortification of yeast fractions significantly reduced disease infection in the challenged breeders. In the same study, the positive culling rate of the pullets and their body weight were significantly reduced because of the addition of yeast fractions to the feed of birds challenged with *Salmonella* infection [14]. The intestinal microflora balance has also been shown to be improved in birds with dietary supplementation of yeast, due to the presence of mannan-oligosaccharides and fructo-oligosaccharides in the cell wall of yeast [15,16,17,18,19]. However, debate remains regarding the effects of active yeast on the production performance and immune status of chickens, particularly when they are fed diets containing different nutrient profiles or are placed under environmental stress [20,21,22,23]. Hayat et al. [24] suggested that this could be due to genetic differences, or differences in species, age, or environmental conditions. Thus, this study was carried out to fill this knowledge gap.

The current study aimed to fill the gap in the literature about the response of broilers fed two types of diets, with or without different concentrations of SC (active yeast) with respect to antioxidant status, blood constituents and immune status. Although previous studies investigated the effect of SC on the productive performance parameters in broiler chickens, there are relatively limited data in the literature on the direct effect of SC probiotic on the antioxidative status, blood constituents and immune status in broiler chickens fed low-density diet. The current literature does not yet adequately address the approaches of nutrient manipulation in broiler feed rations to shed light on the relationship between the effect of SC in low-density diets and the immune response of broiler chickens. Accordingly, this study was executed to elaborate on this vital relationship.

## 2. Materials and Methods

### 2.1. Birds, Dietary Treatments, Experimental Plan and Housing

This research work was approved by the Deanship of Scientific Research, King Abdulaziz University, Saudi Arabia, under protocol no: (FP-217-42 H). The protocol recommends general humane treatment of animals that did not cause animal (s) pain, suffering, distress, or lasting harm, according to the Royal Decree number M59 in 14/9/1431H.

A total of 216, 1-day-old Arbor Acres broilers (mixed sexes) were acquired. The chicks were marked randomly by way of wing-banding and were housed in 36 pens with 6 birds per pen (replicate). Each treatment involved 6 replicates. The body weight (BW) of all chicks was similar at the start of the experiment.

The chickens were provided mash feeds ad libitum, along with freely accessed waterers. During the first 7 days, 23 h of light were used, followed by 20 h of light until the end of the experiment.

A factorial design (2 × 3) was applied to the experiment using two diets (a regular versus a low-density diet containing 10% fewer nutrients than the regular diet) and three levels of SC (an unfortified standard, 0.02%, or 0.04% SC). The broiler feed rations were formulated based on the Arbor Acres broilers guide [25]. There were six replications in each treatment and each replicate involved six birds. The SC yeast was purchased from China Way Co-operation, Taiwan, and had 12,000,000,000 active yeast per gram. The optimal dosage of SC was 200 to 400 g per ton of feed. Table 1 shows the composition of the dietary treatments fed to the chickens.

### 2.2. Data Gathering

Average pen body weight (g) was recorded at 1, 21 and 38 days of age and used to calculate the body weight gain (BWG, g/bird). The average pen feed intake (g/bird) was recorded and used to calculate the feed conversion rate (FCR, g feed/g gain) and survival rate (100–mortality rate) during the following periods: 1–21 days, 22–38 days and 1–38 days of age. The production index was calculated as follows: BW (kg) × survival rate Production index = ×100 production period in days × FCR.

### 2.3. Blood Sampling

Blood was collected from each treatment group (*n* = 6) before vaccination and again at 8 days post-vaccination. The serum was harvested by centrifuging the blood at 1500× *g* for 15 min.

### 2.4. Antioxidant Status and Biochemical Traits

Serum total antioxidant capacity (TAC) and malondialdehyde (MAD) were assayed as described in Erel [26] and Wyatt et al. [27], respectively. They were determined using commercial kits produced by Diamond Diagnostics (23 EL-Montazah St. Heliopolis, Cairo, Egypt, http://www.diamonddiagnostics.com (accessed on 1 February 2022). Total plasma protein and albumin concentrations were measured using the methods outlined in Armstrong and Carr [28] and Doumas and Peters [29], respectively. Subtracting albumin concentration from serum total protein gives an estimate of the globulin concentration, as described in Giangiacomo et al. [30]. Various kinds of globulin (α-, β- and γ-globulin) were determined based on methods described in Elias [31]. The activities of alanine aminotransferase (ALT, U/L) and aspartate aminotransferase (AST, U/L) were determined using techniques described in Reitman and Frankel [32]. Alkaline phosphatase (ALKP) enzyme action was measured in plasma, as described by Kim and Wyckoff [33].

### 2.5. Hematological Parameters

Wintrobe hematocrit tubes were used to measure the packed cell volume (PCV, %) by centrifugation for 20 min. at 2000× *g*. Hemoglobin (Hgb) level was estimated using the technique described in Eilers [34]. The mean corpuscular volume (MCV, µm 3), mean corpuscular hemoglobin (MCH, Pg) and mean corpuscular hemoglobin concentration (MCHC, g/dL) were measured using the equations described in Jain [35].

### 2.6. Immune Indices

The phagocytic activity (PA, % of phagocytic cells engulfing yeast cells) and phagocytic index (PI, number of yeast cell phagocytized/number of phagocytic cells) were determined as described in Kawahara et al. [36].

Broiler chickens were vaccinated according to the following schedule: inactivated avian influenza (AI) H5N2 at 10 days of age. Chickens were vaccinated with clone 30 eye drop on day 8 for Newcastle disease (NDV) and bivalent NDV vaccine was administered underneath the neck membrane, simultaneously with clone 30, at 8 days of age. The Gumboro intermediate vaccine and clone 30 were administered at 12 and 21 days of age, respectively (Nobilis, Intervet, Boxmeer, The Netherlands).

Blood samples (*n* = 6 per group) were taken just before vaccination (0 days post-vaccination) and again on 8th day post-vaccination. The samples were centrifuged at 1500× *g* for 15 min for serum separation, to determine antibody titers against NDV via the hemagglutination inhibition test (HI) test. This test was done using hemagglutination inhibition (HI) test according to OIE [37]. The geometric mean titer was calculated as recommended by the World Organization for Animal Health (OIE) [38].

Antibody responses were determined by the HI test, according to Seal et al. [39]. The assay measures antibodies attached to influenza antigen-coated plates [40]. Hemagglutination inhibition for NDV and AI were measured as described in Takatsy and Hamar [41].

A lymphocyte transformation test was performed, as described in Balhaa et al. [42]. Lympholyte-H (Cedarlane Laboratories Ltd., Burlington, ON, Canada) was used to layer the collected heparinized blood. After centrifugation, the lymphocytes in the interface layer were collected, washed and suspended in culture medium.

Serum bactericidal activity to the *Aeromonas hydrophila* strain was conducted following the protocols described in Rainger and Rowley [43]. The turbidimetric method was used to measure serum lysozyme activity [44]. The results were reflected as one unit of lysozyme activity as a reduction in absorbency of 0.001/min Lysozyme activity = (A0 − A)/A.

### 2.7. Challenge Test

The challenge test was conducted to study the impact of the diet on the survival rate of chicks [45,46] between 38 and 48 days of age. Six broiler chickens per treatment were randomly selected at 38 days of to represent all treatment replications. The chickens were vaccinated with inactivated avian influenza (AI) H5N2 at 10 days of age and then challenged with H5N1 at 38 days via the oculo-nasal route with 0.2 mL/bird (10^6^/dose). The H5N1 was from research laboratory of Poultry Disease, Fac. Vet. Med., Damanhour University, where the challenge test was carried out following the regulations for animal welfare approved by the authorized ethics committee of the Egyptian Ministry of Agriculture according to Decree No. 27, 1967. The mortality was recorded daily during 38–48 days of age.

### 2.8. Histopathological Study

On day 38, 6 chickens from each treatment replicate were randomly selected and euthanized under anesthesia via an intravenous injection of sodium pentobarbital (50 mg/kg; CAMEO chemicals, Tampa, FL, USA). Necropsies were performed for sample collection. Lymphoid organs (bursa of Fabricius, thymus and spleen) were weighed, and the body weight ratios of organs were calculated.

In addition, intestine, bursa of Fabricious, thymus and spleen specimens were collected from randomly collected broilers (*n* = 6 per treatment) at 38 days of age. Tissue specimens were prepared as previously described by Culling [47].

### 2.9. Morphometrical Study

An Optika binocular microscope, with an Optika imaging analyzer, was used to examine the morphological appearance of intestinal villi, determine the absorption surface and measure the longitudinal axis of the large follicle of the bursa. Five segments from each bird were used for this examination. In addition, quantitative measurements of the thymus cortical: medullary ratio were performed and the hyperplasia of the lymphoblastic cells was assayed by examining the spleen. The scale used was as follows: (−) for weak hyperplasia; (+) for moderate hyperplasia; (++) for severe hyperplasia.

### 2.10. Statistical Analyses

The data were analyzed using general linear models in SAS (SAS Institute, Cary, NC, USA [48]). A two-way factorial design (two kinds of diets × three concentrations of SC) was used to analyze the effects of the treatments on most of the parameters. An exception was survival rate in the challenge study, where age was included as a main effect only. The replicate was the experimental unit. Data were arcsine transformed prior to analysis to improve normality. Student–Newman–Keuls (SNK) post hoc tests were applied to evaluate differences between factor levels in the model. Differences were considered significant if *p* ≤ 0.05.

## 3. Results

### 3.1. Growth Performance

Table 2 shows the impact of yeast concentrations on body weight and the European Production Efficiency Index (EPEI) of broilers fed regular- and low-density diets. During the experiment, the low-density diet was found to decrease the final body weight and EPEI, reaching 5% over the duration of the study. Diets supplemented with 0.02% and 0.04% SC resulted in a production index that was significantly enhanced relative to the control diet. However, the addition of SC in the diet at a concentration of 0.04% had a more substantial effect on the growth of 38-day-old chickens than the 0.02% level (Table 2). There was no significant relationship between the amount of SC and diet on the growth of broilers or EPEI between 1 and 38 days of age.

### 3.2. Antioxidant Status and Biochemical Traits

Table 3 and Table 4 show the impact of different yeast concentrations on the liver enzyme index, peroxidation index and blood serum biochemical constituents of broilers fed regular and low-density diets from 1 to 38 of age.

Data for the biochemical components of blood serum show no significant impact of diet type on serum biochemical constituents (total protein, albumin, α-, β-globulin, globulin and globulin/albumin ratio). However, γ-globulin, AST and ALT were significantly lower in birds fed a low-density diet than a regular-density diet (Table 3 and Table 4). Supplementation of the diet with SC at 0.02 g/kg and 0.04% significantly lowered serum AST, ALT and MAD relative to the standard. Additionally, total serum protein and β- globulin were significantly greater in the groups that received SC supplementation compared with control groups without SC. There was no interaction effect between SC level and dietary treatment on blood biochemical constituents (ALT/AST and alkaline phosphatase, Table 3; and total protein, albumin, α, β and δ- globulin, G/A ratio, Table 4). However, there was a significant impact of the interaction on serum AST, ALT and MAD. Supplementation with SC at both concentrations significantly decreased serum AST and ALT of broilers on the low-density diet compared to the regular-density diet. Additionally, 0.02% of SC decreased MAD in broilers fed a low-density diet compared to a regular density diet.

### 3.3. Hematology of Blood

Table 5 shows the effects of different concentrations of yeast on white blood cells and its subpopulations of broilers fed regular- and low-density diets from 1 to 38 days of age. The results showed that lymphocytes of broilers fed a low-density diet were significantly higher than that of broilers fed a regular-density diet, but eosinophils were lower. The addition of SC to the diet greatly enhanced PCV, lymphocytes and monocytes; and 0.02% SC significantly increased Hgb and RBCs, but decreased heterophils and the H/L ratio, relative to control diet (Table 5). However, the addition of SC to the diet at 0.04% significantly increased heterophils.

There were significant effects of interaction between dietary treatments and SC concentrations on PCV, Hgb, MCV, MCH, white blood cells (WBCs) and the H/L ratio. Results showed that SC supplementation increased PCV and Hgb for birds fed a regular-density diet, but 0.4 g/kg SC significantly decreased Hgb in birds fed a low-density diet. In addition, MCV and MCH were increased dramatically by the addition of 0.04% SC to the regular-density diet, but they were reduced in birds fed the low-density diet. The results showed that using 0.02% and 0.04% of SC increased WBCs in birds fed a regular-density diet, but 0.04% of SC significantly decreased WBCs in those fed a low-density diet. On the other hand, 0.02% of SC significantly decreased H/L in birds fed the low-density diet only (Table 5).

### 3.4. Lymph Organs and Immune Response

Table 6 shows the effects of the experimental treatments on the lymphoid organs of broilers. The results showed no significant effect of diet density on lymph structures like the spleen, absolute weight of thymus and bursa of Fabricius. However, the percentage of thymus was significantly greater in birds fed a low-density diet than that in birds fed a regular-density diet. Immune responses to NDV and AI, as measured by HI, were not influenced by diet type. These organs, as well as the immune response to NDV and AI, were significantly greater in broilers fed a diet supplemented with 0.02% or 0.04% of SC than those fed a diet without SC supplementation. Moreover, the effects on the thymus, bursa of Fabricius and NDV and AI showed stepwise increases. There were significant effects of interactions between diet type and SC on the percentage of spleen weight, bursa weight and immune response to NDV. The results indicated that the absolute weights of the spleen and bursa of Fabricius significantly decreased in the group fed the regular-density diet supplemented with 0.04% of SC. Still, both levels of SC significantly increased immune response to NDV. On the other hand, both concentrations of SC significantly increased the absolute weights of the spleen and bursa of Fabricius in birds fed the low-density diet, but the response to NDV was stepwise (Table 6).

### 3.5. Immune Indices

Table 7 and Table 8 show the impacts of the different experimental treatments on the immune parameters and survival rate of broilers, respectively. Diet type significantly influenced LYS, TAC and PA, revealing an increasing effect of a low-density diet on immune parameters, but lower TAC (Table 7).

Supplementation of the diet with SC significantly decreased TAC, but enhanced PI relative to the standard diet and had no impact on other traits related to the immunity, including serum LTT, BACT, LYS and PA (Table 7). In addition, the survival rate of broiler chickens fed a diet fortified with 0.04% of SC was significantly greater than that of chickens fed a dietary supplement of 0.02% SC, or a diet without SC (Table 8).

The interaction between SC concentration and diet type did not influence the LTT, BACT, LYS, TAC, PI, PA, or survival rate during the challenge experiment (Table 7 and Table 8).

### 3.6. Histology Study

**Figure 1:**
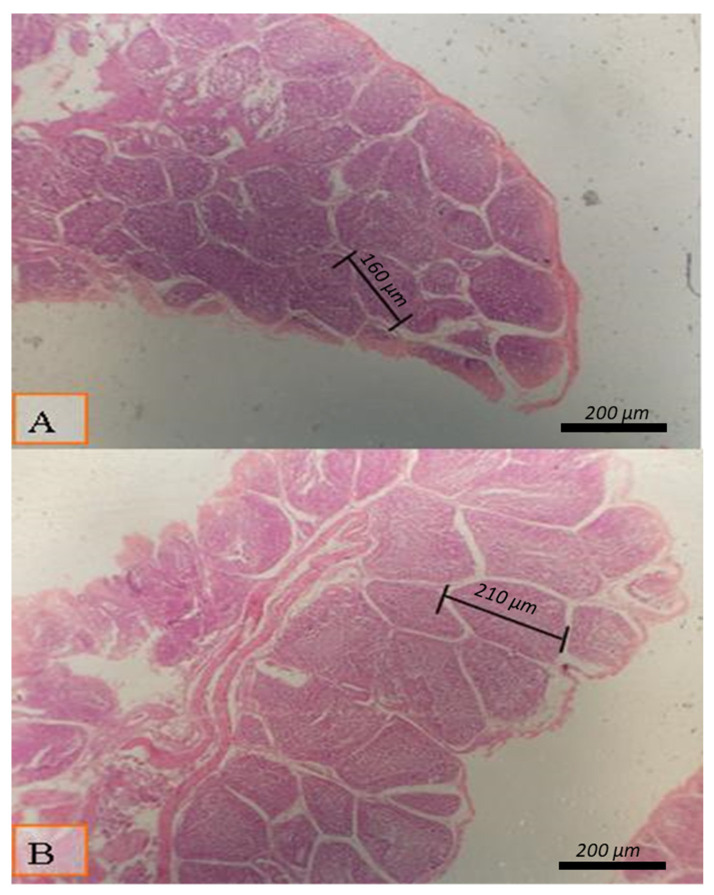
Micrograph of bursa of Fabricious of broiler at day 38 of age stained with HandE (X40) to investigate the follicle diameter in different groups; the distance between two follicular polar as presented all groups by lines: (**A**) Broilers supplemented with 0.02% *Saccharomyces cerevisiae*;(**B**) broilers supplemented with 0.04% *Saccharomyces cerevisiae*. Moderate enhancement in the follicular diameter was detected in broilers supplemented with 0.04% *Saccharomyces cerevisiae* (**B**).

**Figure 2:**
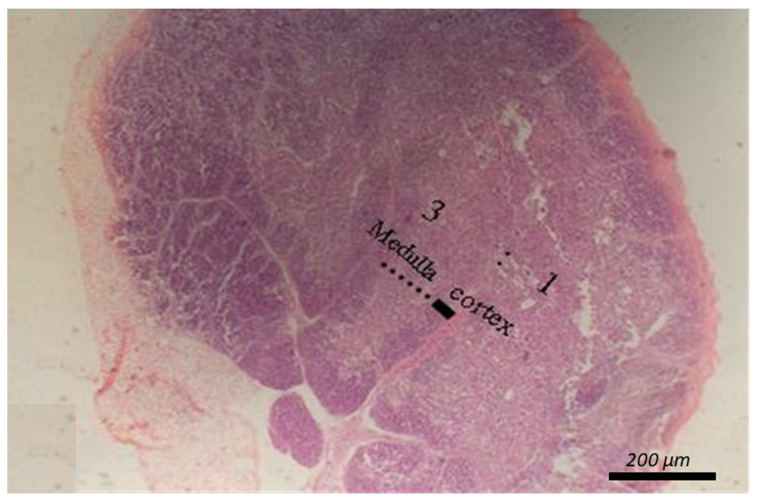
Micrograph of the thymus stained with HandE (X40) to explore the thymic cortical: medullary ratio.

**Figure 3:**
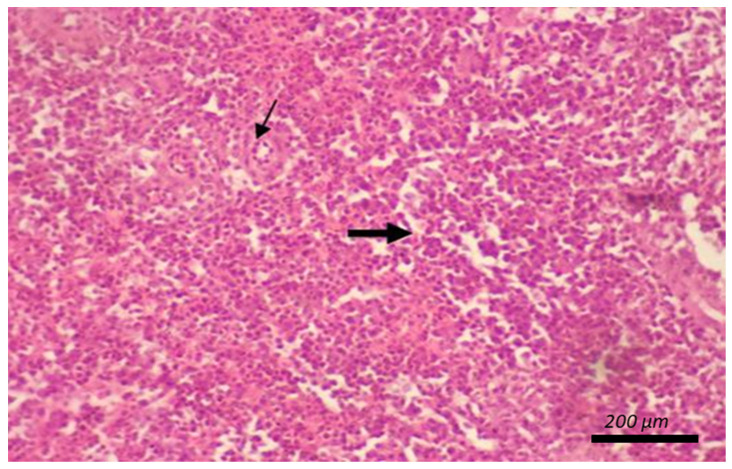
Micrograph of the spleen stained with HandE (X40) of regular density diet presented normal splenic histology featuring splenic arteriole (thin arrow) with white and red pulp (thick arrow). All groups presented the normal splenic histology as the control.

Table 9 shows the impact of diet type and SC concentration on the morphology of the intestines and bursa of Fabricius and spleen, as shown in Figure 1, Figure 2 and Figure 3. Diet type did not influence the length of intestinal villi or diameter of the large follicle of the bursa of Fabricius (Table 9). No changes were seen in the spleen and thymus due to diet type, SC concentration, or the interaction between these terms (Table 9; Figure 2 and Figure 3).

Supplementation of the diet with 0.04% SC significantly enhanced the length of intestinal villi (Table 9) and the diameter of the large bursal follicle (Table 9 and Figure 1). The intestinal villi and bursa of Fabricious were enhanced by 29.8% and 22.9%, respectively. Both traits were significantly enhanced with the supplementation of SC at a concentration of 0.02%. The large bursal follicle’s intestinal villi length and diameter were not affected by the interaction between diet type and SC concentration.

## 4. Discussion

The present work was conducted to fill the gap in knowledge regarding the impact of active SC yeast in relation to dietary composition on the antioxidant status, blood constituents and immune response of broiler chickens. Adding an SC product to the feed at concentrations of 0.02% or 0.04% improved the performance of broilers from 1 to 38 days of age.

To our knowledge, this is the first study investigating the interactive relationship between using yeast in low-density diets and its effect on immune response in broiler chickens. The percentage of thymus was significantly greater in birds fed a low-density diet than that of birds fed a regular-density diet. The immune response to NDV and AI were significantly greater in broilers fed a diet supplemented with 0.02% or 0.04% of SC than those fed a diet without SC supplementation. In addition, there were significant interactions between diet type and SC on the percentage of spleen weight, bursa weight and immune response to NDV. The results indicated that the absolute weights of the spleen and bursa of Fabricious were significantly high in the group fed the low-density diet supplemented with 0.04% of SC. Still, both levels of SC significantly increased immune response to NDV. Interestingly, both concentrations of SC significantly increased the absolute weights of the spleen and bursa of Fabricious in birds fed the low-density diet.

The increase in feed cost accompanied by the reduction in the availability of corn as a main feed ingredient will affect the production efficiency of poultry on the global level, especially during global pandemics such as the current coronavirus crisis. Nutritional manipulation by using the low-density diet supplemented with yeast could provide a great opportunity to improve the economic outcome by reducing the feed cost, which constitutes approximately 60–70% of the total poultry operation cost. Using low density-diet in broiler rations will provide a positive alternative to reduce feed cost. The other side of the coin is that using yeast in these diets compensated for the low-density contents of the diets by improving the antioxidant status and immune response of the broiler chickens. Any improvement in nutrition management and feed cost will have a direct impact on profitability and efficiency of poultry industry [49,50].

In addition, the group treated with 0.04% of SC had a significantly higher immune response than the other groups. These results contribute to the poultry industry important information that will improve production efficiency.

In addition, 0.02% of SC decreased MAD of broilers fed a low-density diet compared with a regular-density diet. These results indicate that SC enhances the oxidative status of broilers. Interestingly, Czech et al. [11] revealed that using 3% of *Yarrowia lipolytica* or SC yeast, in combination with *Bacillus* sp. probiotic, in the feed of turkeys from 7 to 112 days of age improved the antioxidant status of birds by preventing lipid peroxidation. This effect enhances the ability of poultry to handle stress and infectious agents. In another study [10], the authors investigated the mechanism by which SC enhances the oxidative status of broiler chickens. The authors included SC in either the feed or drinking water of stressed broilers and measured CYP1A2 and melanocortin-2 receptor (MC2R) gene expression in the adrenal glands and IL10 and AvBD1 in the spleen. The authors concluded that using SC in broilers’ feed or drinking water for 40 days decreased stress and MC2R gene expression. They also showed that supplementation of SC fermentate in the feed was marginally more effective than adding it to drinking water in stimulating oxidative status and reducing stress in broiler chickens [10] and in detoxifying nitrate (21, 22) and aflatoxin (22, 23).

There has been an interest in using a low-density diet to feed broilers, to lower the growing pressure on the skeletal system of the bird and decrease skeletal diseases, the cost of feed and environmental pollution [51,52].

The body weight and EPEI were significantly decreased by 7.8% and 5.4%, respectively, over the study period for birds on the low-density diet compared to the regular-density diet. Indeed, the main effect of regular diet under the three SC levels was 2099 g while the mean body weight of the control diet without SC supplementations (1946 + 1784) was 1965 g. These findings indicate that the negative impact of diet structure persisted during the experimental period from 1 to 38 days of age [53].

The outcomes showed that the low-density diet improved liver function and increased the percentage of thymus and lymphocytes and PA, but decreased γ-globulin, eosinophils, TAC, ALT and AST. The current study revealed that supplementation of the feed with SC at 0.02 g/kg and 0.04% significantly lowered serum AST, ALT and MAD relative to the standard diet. Gheisari and Kholeghipour [9] showed that using live yeast had no significant impact on hematological indices such as RBCs, WBCs and PCV. On the other hand, in another study, there was a positive association between supplementation of feed with SC and hematological traits of chickens, such as RBCs, WBCs and PCV [51]. In the same study, probiotics had no effect (*p* > 0.05) on hemoglobin and WBCs at the finisher phase. Yet, a significant effect (*p* < 0.05) was observed for RBCs and packed cell volume.

Gut morphology was modulated because of the addition of 10% wheat bran in the low-density diet. The results indicated that the diets had no impact on the length of intestinal villi. Previous studies have shown that dietary cereal with a high nonstarch polysaccharides (NSP) level could enhance the dimension of the gastrointestinal tract [54]. Steenfeldt [55] observed that the arabinoxylan level in wheat is significantly and positively correlated to the relative masses of the duodenum, jejunum and ileum. It has been stated that dietary supplementation may modulate the morphology of the intestinal mucosa. Accordingly, NSP in the diet can also impact the morphology of the gastrointestinal tract [56]. Iji [57] showed that the crypt deepness of the jejunum and ileum was significantly enhanced by dietary addition of guar gum and xanthin gum. This finding demonstrated that NSP can improve cell turnover in the gastrointestinal tract. Enhanced crypt deepness indicates enhanced villus cell proliferation and in turn improved utilization of the nutrients by the gastrointestinal tract. This suggests that these cereals impact the mass of the gastrointestinaltract and morphology of the intestine [58].

These results suggest that the dilution of nutrients in feed via adding 10% wheat bran improves the immune response and production index of broilers 1 to 38 days of age. These outcomes agree with earlier studies by Abudabos [53] and Attia [54]. Wheat bran polysaccharides have been shown to act as antioxidants and immunostimulators and have anti-inflammatory, antitussive, anticancerous and antimutagenic properties [59,60,61,62]. Furthermore, wheat bran arabinoxylans have been shown to to enhance macrophage phagocytosis in animals [63]. They are immunostimulants of the antibody response in chickens by enhancing the total IgG and IgM anti-SRBC antibody titers on 7th and 14th day post primary antibody response (PPI) and post-secondary inoculation (PSI) of sheep red blood cells (SRBCs) compared to the control. Additionally, Korte et al. [64] stated that supplementing feed with arabinoxylans significantly induced anti-SRBC antibody titers, representing enhanced humoral immunity in chickens.

The results indicated that supplementing feed with SC at either 0.02% or 0.04% significantly affected growth and EPEI relative to the control dietary treatment. The effect persisted throughout all tested periods. Furthermore, supplementing the low-density diet with 0.02% or 0.04% of SC resulted in an enhanced production index compared with the regular-density diet lacking the addition.

These results agree with other studies that have investigated similar effects of SC on growth performance [65,66,67,68]. In addition, the positive effect of a higher dose of SC are in line with results reported by Valdivie [69], who found that the growth performance of broilers significantly improved with an increased supplementation dose of SC. The improved gastrointestinal health and growth performance of broilers supplemented with SC may be due to the presence of effective ingredients in SC such as Vitamin B, cellulostic enzymes, phytase, monooligosaccharides (MOS) and glucomannan [70].

A diet supplemented with 0.04% SC increased the length of the villi and SC supplementation enhanced the production index and the body weight of broiler from 1 to 38 days of age in a dose-dependent manner.

There were significant improvements in blood serum biochemistry and liver function due to SC supplementation. Consistent with these results, Paryad and Mahmoudi [71] and Hosseini [72] showed that SC at 1.5% significantly enhanced total plasma protein, albumin and globulin and WBCs and decreased the H/L ratio. Furthermore, Zhang et al. [64] revealed that SC supplementation to broiler chickens significantly lowered the 2-thiobarbituric acid-reactive substances (TBARS) in the breast and drumstick meats and increased villus height, compared to the control group.

Supplementation of SC significantly enhanced the spleen, thymus, bursa of Fabricious and HI in response to NDV and AI, with a positive concentration-dependent impact of SC on the thymus, bursa of Fabricious and immune response to NDV and AI. The diameter of the bursal follicle significantly enhanced at 0.04% SC, indicating an improvement in the number of B-lymphoblasts, leading to an increase in the B-lymphocytes responsible of humoral immunity stimulation through antibody production. Further evidence of this effect was reflected by the increased survival rate of broilers challenged with AI at 38 d of age. In addition, SC supplementation was associated with improvements in β-globulin and hematological traits such as PCV, Hgb, RBCs, lymphocytes, monocytes and PI. These data provide more evidence for an improved health status of broilers fed a diet supplemented with SC. The effect of SC on the relative weights of the thymus and bursa and immune response to NDV and AI, was dose-dependent. Similarly, Newman [73], Spring et al. [74] and Zhang et al. [70] showed that SC supplementation of the diet enhanced production performance by improving the immune status, intestinal lumen health and digestion and nutrient utilization of birds. In addition, Gheisari and Kholeghipour [9] found that broiler chickens fed SC at a concentration of 0.02% had higher antibody titers against NDV than the control at 38 d of age, but it did not affect AI titers. The positive effect of SC on immune response could be attributed to its cell wall constituents, including chitin, mannan and glucan, which have immunostimulant effects [2,71,75,76,77,78].

## 5. Conclusions

The antioxidant status and total antioxidant capacity of broiler chickens were improved by supplementation of the diet with SC. Supplementation of either a regular-density diet or a low-density diet with SC at either 0.02% or 0.04% enhanced the BWG and EPEI of broilers during 1 to 38 days of age. Additionally, broilers fed a low-density diet supplemented with either 0.02% or 0.04% SC had a greater body weight and EPEI than birds fed the control diet with no supplementary SC. However, fortification of the diet with 0.04% resulted in significantly enhanced immune organs and a higher immune response.

## Figures and Tables

**Table 1:** Basal chemical composition of the experimental diets.

Ingredients (%)	Regular-Density Diet			Low-Density Diet		
	Starter	Grower	Finisher	Starter	Grower	Finisher
Maize	51.27	52.02	55.85	46.01	46.61	50.40
Rye	0	5	7	0	4.48	6.2
Wheat bran	0	0	0	10	10	10
Soybean meal (44% CP)	32.8	24.4	28.0	29.3	21.9	25.0
Vegetable oil	2.25	2.0	5.3	2.01	1.79	4.7
Full-fat soybean meal	10	13	1.6	9	11.64	1.42
Dicalcium phosphate	1.8	1.6	1.0	1.8	1.6	1.0
Limestone	1.0	1.0	0.15	1.0	1.0	0.15
L-Lysine	0.10	0.15	0.2	0.10	0.15	0.2
DL-Methionine	0.15	0.20	0.30	0.15	0.20	0.30
Vit + min premix ^1^	0.30	0.30	0.30	0.30	0.30	0.30
NaCl	0.30	0.30	0.30	0.30	0.30	0.30
Washed building sand	0.03	0.03	0.00	0.03	0.03	0.03
Total	100	100	100	100	100	100
Calculated and deterimed analyses						
Metabolizable Energy (kcal/kg) ^2^	3038	3100	3196	2735	2790	2876
Crude Protein (%) ^3^	22.8	21.2	18.5	20.5	19.1	16.7
Lysine (%) ^2^	1.33	1.23	1.04	1.20	1.11	0.94
Methionine (%) ^2^	0.50	0.52	0.48	0.45	0.47	0.43
Meth + cysteine (%) ^2^	0.87	0.87	0.78	0.78	0.78	0.70
Calcium (%) ^2^	0.91	0.85	0.83	0.82	0.77	0.75
Av. P (%) ^2^	0.46	0.41	0.41	0.414	0.369	0.369
Crude fat (%) ^3^	6.09	6.45	6.8	5.48	5.81	6.12
Crude fibre (%) ^3^	3.55	4.48	4.75	3.20	4.03	4.28
Ash (%) ^3^	5.22	5.48	5.71	4.70	4.93	5.14

^1^ Vit + Min mix. contains the following nutrients. Values are per kilogram of the diet: Vit. A, 12,000 IU; Vit. E (dl-α-tocopheryl acetate), 20 mg; menadione, 2.3 mg; Vit. D_3_, 2200 ICU; riboflavin, 5.5 mg; calcium pantothenate, 12 mg; nicotinic acid, 50 mg; Choline, 250 mg; Vit. B_12_, 10 μg; Vit. B_6_, 3 mg; thiamine 3 mg; folic acid, 1 mg; d-biotin, 0.05 mg; Trace minerals (mg/kg of diet): Mn, 80; Zn, 60; Fe, 35; Cu, 8; Selenium, 0.1 mg, ^2^ Calculated analyses, ^3^ Determined analyses.

**Table 2:** Impact of different concentrations of *Saccharomyces cerevisiae* on body weight, survival rate, European Production Efficiency Index and blood hematological parameters of broilers fed regular- or low-density diets from days 1 to 38 of age.

Treatment		Body Weight at 38 Days of Age, g	Survival Rate, %	European Production Efficiency Index	PCV, %	Hemoglobin, g/dL	RBC, 10^6^/uL	MCV, fL	MCH, pg	MCHC, g/dL
Effect of diet										
Regular density		2099 ^a^	100	314 ^a^	30.4	9.91	1.65	186	60.6	32.7
Low density		2004 ^b^	100	297 ^b^	30.0	10.0	1.72	177	58.7	33.3
Effect of yeast concentration										
Control		1865 ^c^	100	281 ^b^	28.3 ^b^	9.43 ^b^	1.60 ^b^	180	59.7	33.2
0.02%		2106 ^b^	100	318 ^a^	31.6 ^a^	10.5 ^a^	1.76 ^a^	182	60.8	33.5
0.04%		2183 ^a^	100	318 ^a^	30.7 ^a^	9.87 ^b^	1.70 ^ab^	182	58.5	32.2
Interaction between diet type and yeast concentration										
Regular density	Control	1946	100	288	18.7 ^b^	8.75 ^b^	1.58	172 ^b,c^	55.7 ^b,c^	32.4
	0.02%	2171	100	321	32.0 ^a^	10.6 ^a^	1.75	185 ^a,b,c^	61.6 ^a,b^	33.4
	0.04%	2179	100	334	32.3 ^a^	10.3 ^a^	1.62	202 ^a^	64.5 ^a^	32.2
Low density	Control	1784	100	274	29.7 ^a^	10.1 ^a^	1.61	188 ^a,b^	63.8 ^a,b^	34.0
	0.02%	2041	100	315	31.3 ^a^	10.5 ^a^	1.77	179 ^a,b,c^	60.0 ^a,b^	33.5
	0.04%	2187	100	303	29.1 ^a^	9.37 ^b^	1.78	163 ^c^	52.4 ^c^	32.3
SEM		41.30	ND	9.67	0.955	0.316	0.058	9.99	3.09	1.02
*p* value										
Diet type		0.0008	ND	0.0408	0.6336	0.7485	0.1473	0.256	0.466	0.458
Yeast		0.0001	ND	0.0006	0.0040	0.0038	0.0273	0.966	0.753	0.442
Interaction		0.1182	ND	0.4298	0.0121	0.0021	0.4088	0.029	0.009	0.715

^a,b,c^ Means within a column with different superscripts are significantly different based on Student–Newman–Keuls (SNK) post hoc tests. MCV = Mean corpuscular volume; MCH = Mean corpuscular hemoglobin; MCHC = Mean corpuscular hemoglobin concentration; Number of observations was 6 replicates per interaction cell. ND = Not done.

**Table 3:** Impact of different concentrations of *Saccharomyces cerevisiae* on liver enzymes and malondialdehyde (MDA) of broilers fed regular- and low-density diets from days 1 to 38 of age.

Treatment		AST (U/L)	ALT (U/L)	AST/ALT Ratio	Alkaline Phosphatase (U/L)	MDA.m Mol/dL
Effect of diet						
Regular density		64.7 ^a^	56.4 ^a^	1.14	11.4	1.44
Low density		63.7 ^b^	55.1 ^b^	1.15	11.6	1.45
Effect of yeast concentration						
Control		66.0 ^a^	57.8 ^a^	1.14	11.5	1.57 ^a^
0.02%		63.43 ^b^	54.9 ^b^	1.15	11.2	1.32 ^c^
0.04%		63.2 ^b^	54.6 ^b^	1.15	11.8	1.45 ^b^
Interaction between diet and yeast concentration						
Regular density	Control	65.5 ^a,b^	57.1 ^a,b^	1.14	11.6	1.41 ^b^
	0.02%	64.2 ^b^	56.1 ^b^	1.14	11.2	1.43 ^b^
	0.04%	64.5 ^b^	56.1 ^b^	1.14	11.3	1.47 ^b^
Low density	Control	66.6 ^a^	58.6 ^a^	1.13	11.3	1.73 ^a^
	0.02%	62.6 ^c^	53.7 ^c^	1.16	11.2	1.21 ^c^
	0.04%	62.0 ^c^	53.1 ^c^	1.16	12.2	1.42 ^b^
SEM		0.537	0.701	0.015	0.587	0.058
*p* value						
Diet		0.0283	0.0299	0.4385	0.6668	0.7284
Yeast concentration		0.0001	0.0001	0.5854	0.6351	0.0005
Interaction		0.0046	0.0052	0.5946	0.6077	0.0001

^a,b,c^ Means within a column with different superscripts are significantly different based on Student–Newman–Keuls (SNK) post hoc test. ALT = Alanine aminotransferase; AST = Aspartate aminotransferase; AST/ALT = Aspartate aminotransferase to alanine aminotransferase ratio; MAD = Malondialdehyde. Number of observations was 6 per interaction cell.

**Table 4:** Impact of different concentrations of *Saccharomyces cerevisiae* on the biochemical constituents of blood serum of broilers fed regular- and low-density diets from days 1 to 38 of age.

Treatment		Serum Biochemical Constituents (mg/dL)						
		Total Protein, g/dL	Albumin, g/dL	Globulin, g/dL	α -Gloulin, g/dL	β -Globulin, g/dL	γ -Globulin, g/dL	GAR
Effect of diet								
Regular density		5.00	2.70	2.24	0.912	0.730	0.595 ^a^	0.846
Low density		4.80	2.73	2.11	0.958	0.720	0.437 ^b^	0.784
Effect of yeast concentration								
Control		4.73 ^b^	2.64	2.09	0.837	0.656 ^b^	0.600	0.828
0.02%		5.01 ^a^	2.70	2.17	0.956	0.768 ^a^	0.450	0.792
0.04%		5.00 ^a^	2.81	2.26	1.01	0.756 ^a^	0.500	0.824
Interaction between diet and yeast concentration								
Regular density	Control	4.78	2.63	2.15	0.837	0.637	0.675	0.870
	0.02%	4.86	2.75	2.26	0.962	0.800	0.500	0.820
	0.04%	5.10	2.72	2.31	0.837	0.762	0.612	0.846
Low density	Control	4.68	2.65	2.03	0.837	0.675	0.525	0.786
	0.02%	4.86	2.65	2.08	0.950	0.737	0.400	0.763
	0.04%	5.02	2.91	2.22	1.087	0.750	0.387	0.802
SEM		0.137	0.153	0.113	0.072	0.044	0.067	0.075
*p* value								
Diet		0.191	0.792	0.1845	0.4459	0.7356	0.0066	0.3243
Yeast		0.0312	0.5149	0.3136	0.0612	0.0323	0.0908	0.8728
Interaction		0.8182	0.6448	0.9241	0.4702	0.5448	0.6516	0.9641

^a,b^ Means within a column with different superscripts are significantly different based on Student–Newman–Keuls (SNK) post hoc tests. GAR = Globulin to albumin ratio. Number of observations was 6 chicks per interaction cell.

**Table 5:** Impact of different concentrations of *Saccharomyces cerevisiae* on white blood cells (WBC) and its subpopulations in broilers fed regular- and low-density diets from 1 to 38 days of age.

Treatment		WBCs, 10^6^/mm^3^	Lymphocytes, %	Monocytes, %	Basophils, %	Eosinophils, %	Heterophils, %	H/L Ratio
Effect of diet								
Regular density		22.8	41.1 ^b^	10.5	0.583	10.0 ^a^	23.4	0.572
Low density		22.7	42.4 ^a^	10.6	0.583	9.29 ^b^	23.7	0.564
Effect of yeast concentration								
Control		22.8	40.0 ^b^	9.68 ^b^	0.562	9.87	23.1 ^b^	0.583 ^a^
0.02%		23.1	42.9 ^a^	11.3 ^a^	0.625	9.56	23.4 ^b^	0.550 ^b^
0.04%		22.4	42.4 ^a^	10.8 ^a^	0.562	9.56	24.1 ^a^	0.571 ^a,b^
Interaction between diet and yeast concentration								
Regular density	Control	21.7 ^b^	39.7	9.25	0.625	10.6	23.0	0.585 ^a^
	0.02%	23.2 ^a^	41.7	11.5	0.750	9.87	23.7	0.571 ^a^
	0.04%	23.5 ^a^	42.0	11.0	0.375	9.62	23.5	0.560 ^a^
Low density	Control	23.8 ^a^	40.3	10.1	0.500	9.12	23.2	0.581 ^a^
	0.02%	23.0 ^a^	44.1	11.1	0.500	9.25	23.1	0.528 ^b^
	0.04%	21.3 ^b^	42.8	10.6	0.750	9.50	24.8	0.583 ^a^
SEM		0.450	0.543	0.379	0.189	0.300	0.362	0.130
*p* value								
Diet		0.8219	0.0059	0.8939	1.0000	0.0041	0.2689	0.4844
Yeast		0.3213	0.0001	0.0004	0.9304	0.4936	0.0174	0.0453
Interaction		0.0001	0.2324	0.1783	0.2321	0.0815	0.3219	0.0485

^a,b^ Means within a column with different superscripts are significantly different based on Student–Newman–Keuls (SNK) post hoc tests. H/L = Heterophil to lymphocyte ratio. Number of observations was 6 per interaction cell.

**Table 6:** Impact of different concentrations of *Saccharomyces cerevisiae* on immune organs and HI titer (log2) in response to avian influenza and Newcastle disease virus in 38-day-old broilers fed either a regular- or low-density diet from days 1 to 38 of age.

Treatment		Spleen, %	Thymus, %	Bursa of Fabricius, %	HI^2^, Log2	
					NDV	AI
Effect of diet						
Regular density		0.135	0.466 ^b^	0.201	3.36	2.83
Low density		0.148	0.516 ^a^	0.223	3.43	2.04
Effect of yeast concentration						
Control		0.110 ^b^	0.425 ^c^	0.167 ^c^	1.12 ^c^	0.125 ^c^
0.02%		0.153 ^a^	0.490 ^b^	0.213 ^b^	3.54 ^b^	2.69 ^b^
0.04%		0.161 ^a^	0.558 ^a^	0.256 ^a^	5.58 ^a^	4.50 ^a^
Interaction between diet and yeast concentration						
Regular density	Control	0.112 ^c^	0.366 ^c^	0.162 ^b^	1.17 ^d^	0.250
	0.02%	0.136 ^b^	0.486 ^b^	0.189 ^b^	3.92 ^c^	3.37
	0.04%	0.155 ^a,b^	0.545 ^a,b^	0.252 ^a^	5.00 ^b^	4.87
Low density	Control	0.108 ^c^	0.484 ^b^	0.171 ^b^	1.08 ^d^	0.00
	0.02%	0.169 ^a^	0.495 ^b^	0.238 ^a^	3.17 ^c^	2.00
	0.04%	0.167 ^a^	0.570 ^a^	0.260 ^a^	6.17 ^a^	4.12
SEM		0.326	0.195	0.014	0.372	0.605
*p* value						
Diet		0.0669	0.0214	0.0606	0.7161	0.1174
Yeast		0.0001	0.0001	0.0001	0.0001	0.0001
Interaction		0.1060	0.0828	0.2739	0.0393	0.6515

^a,b,c,d^ Means within a column with different superscripts are significantly different based on Student–Newman–Keuls (SNK) post hoc tests. HI = Hemagglutination inhibition test; NDV = Newcastle disease virus; I = Influenza antigen. Number of observations was 6 chicks per interaction cell.

**Table 7:** Impact of different concentrations of *Saccharomyces cerevisiae* on immune indices of broilers fed a regular- or low-density diet from 1 to 38 days of age.

Treatment		LTT,%	BACT,%	LYS,%	TAC, mMol/dL	PI	PA,%
Effect of diet							
Regular density		24.2	42.1	0.082 ^b^	429 ^a^	1.58	17.4 ^b^
Low density		25.8	43.2	0.098 ^a^	419 ^b^	1.53	18.8 ^a^
SEM		0.599	0.527	0.004	2.19	0.025	0.375
Effect of yeast concentration							
Control		24.8	43.9	0.081	436 ^a^	1.46 ^b^	17.5
0.02%		25.1	41.8	0.095	420 ^b^	1.61 ^a^	18.3
0.04%		25.0	42.3	0.095	416 ^b^	1.60 ^a^	18.6
SEM		0.733	0.645	0.005	2.68	0.031	0.460
Interaction between diet and yeast concentration							
Regular density	Control	23.6	43.2	0.071	443	1.45	16.0
	0.02%	24.3	41.2	0.082	425	1.61	17.8
	0.04%	24.7	42.0	0.092	419	1.68	18.5
Low density	Control	26.1	44.6	0.091	430	1.47	19.0
	0.02%	26.0	42.5	0.107	414	1.61	18.8
	0.04%	25.3	42.7	0.097	413	1.52	18.7
SEM		1.03	0.913	0.007	3.39	0.044	0.650
*p*-value							
Diet		0.0693	0.1394	0.0128	0.0027	0.2240	0.0111
Yeast		0.9552	0.0746	0.1410	0.0001	0.0026	0.2054
Interaction		0.6676	0.9366	0.4206	0.6813	0.0942	0.1052

^a,b^ Means within a column with different superscripts are significantly different based on Student–Newman–Keuls (SNK) post hoc tests. LTT = Lymphocyte transformation test; BACT = Bactericidal activity; LYS = Lysozyme activity; TAC = Total antioxidant capacity; PI = Phagocytic index; PA = Phagocytic activity. Number of observations was 6 per interaction cell.

**Table 8:** Impact of different concentrations of *Saccharomyces cerevisiae* on the survival rate of broiler chickens between 38 and 48 days of age that were fed a regular- or low-density diet and infected with HPAIV H5N1 at 38 days of age.

Treatment		Survival Rate after Infection of Influenza at 38 of Age,%
Effect of diet		
Regular density		64.8
Low density		59.2
Effect of yeast concentration		
Control		56.0 ^b^
0.02%		56.0 ^b^
0.04%		71.0 ^a^
Effect of age		
39 d (1) challenge		100 ^a^
40 d (2) challenge		100 ^a^
41 d (3) challenge		70.0 ^b^
42 d (4) challenge		56.6 ^b,c^
43 d (5) challenge		50.0 ^b,c^
44 d (6) challenge		50.0 ^b,c^
45 d (7) challenge		50.0 ^b,c^
46 d (8) challenge		45.0 ^b,c^
47 d (9) challenge		40.0 ^c^
48 d (10) challenge		30.0 ^c^
Interaction between diet and yeast concentration		
Regular density	Control	56.0
	0.02%	64.0
	0.04%	70.0
Low density	Control	56.0
	0.02%	48.0
	0.04%	72.0
SEM		6.57
*p* value		
Diet		0.2947
Yeast		0.0001
Interaction		0.1380

^a,b,c^ Means within a column with different superscripts are significantly different based on Student–Newman–Keuls (SNK) test. Number of observations was 6 broilers of 38-day-old per interaction cell.

**Table 9:** Impact of different concentrations of *Saccharomyces cerevisiae* on the morphology of the intestine, bursa of Fabricius and follicular cortical:medullary ratio of the thymus in 38-day-old broilers fed regular- or low-density diet.

Treatment		Length of Intestinal Villi, µm	L. Axis of Large Follicle of Bursa of Fabricius, µm	Follicular Cortical: Medullary Ratio of the Thymus
Effect of diet				
Regular density		239	166	1:3
Low density		248	177	1:3
Effect of yeast concentration				
Control		215 ^b^	157 ^b^	1:3
0.02%		227 ^b^	164 ^b^	1.3
0.04%		289 ^a^	193 ^a^	1:3
Interaction between diet and yeast concentration				
Regular density	Control	211	157	1:3
	0.02%	226	159	1:3
	0.04%	279	181	1:3
Low density	Control	219	157	1:3
	0.02%	227	170	1:3
	0.04%	298	206	1:3
SEM		9.61	8.07	ND
*p* value				
Diet		0.2389	0.0851	ND
Yeast		0.0001	0.0002	ND
Interaction		0.6707	0.2832	ND

^a,b^ Means within a column with different superscripts are significantly different based on Student–Newman–Keuls (SNK) post hoc tests. Number of observations was 6 per interaction cell per age. ND = Not done.

## Data Availability

Data were presented in the manuscript.
